# SASH1 as a Context-Dependent Multi-Docking Scaffold Linking Receptor Signaling to Cytoskeletal Dynamics

**DOI:** 10.3390/ijms27157052

**Published:** 2026-08-06

**Authors:** Christopher M. Clements, Md Saiful Islam Roney, Yiqun G. Shellman

**Affiliations:** 1 Department of Dermatology, University of Colorado Anschutz Medical Campus, Aurora, CO 80045, USA; christopher.m.clements@cuanschutz.edu (C.M.C.); mdsaifulislam.roney@cuanschutz.edu (M.S.I.R.); 2 Department of Biochemistry and Molecular Genetics, University of Colorado Anschutz Medical Campus, Aurora, CO 80045, USA; 3 Gates Institute, University of Colorado Anschutz Medical Campus, Aurora, CO 80045, USA; 4 Rocky Mountain Regional VA Medical Center, Aurora, CO 80045, USA

**Keywords:** SASH1 (SAM and SH3 domain-containing protein 1), scaffold protein, sterile alpha motif (SAM) domain, SRC-homology-3 (SH3) domain, SLy proteins associated disordered region (SPIDER), tankyrase, pigmentation disorders, tumor suppressor, SASH1-associated pigmentary diseases (SAPD)

## Abstract

SASH1 (SAM [sterile alpha motif] and SH3 [SRC-homology-3] domain-containing protein 1) is a multidomain scaffold implicated in pigmentation, innate immunity, receptor signaling, cytoskeletal dynamics, vascular biology, and tumor suppression. Although genetic and expression studies link SASH1 dysfunction to diverse diseases, a unifying mechanistic framework has remained elusive. Here, we synthesize current knowledge of SASH1 structure, interaction networks, and biological functions across cell types and disease contexts. SASH1 contains an intrinsically disordered SPIDER (SLy Proteins Associated Disordered Region), an SH3 domain, two SAM domains, and multiple linear motifs; together, these elements mediate interactions with EphA8 (ephrin type-A receptor 8), β-arrestin 1, TRAF6 (TNF receptor-associated factor 6), CRKL (CRK-like proto-oncogene), IQGAP1 (IQ-motif-containing GTPase-activating protein 1), cortactin, and TNKS2 (tankyrase-2). We propose that SASH1 functions as a context-dependent multi-docking scaffold that organizes signaling architecture. Its modular domains, intrinsically disordered regions, and dual SAM domains enable flexible, multivalent interactions with partners that can be grouped into three functional modules: receptor regulation, intracellular signaling, and cytoskeletal organization. Notably, many SASH1 partners are themselves scaffold or adaptor proteins, allowing integration into pre-existing networks in a hierarchical ‘scaffold-of-scaffolds’ manner. Through selective partner recruitment, SASH1 links cell-surface receptor inputs to downstream signaling pathways and cytoskeletal remodeling. This model provides a mechanistic framework for how SASH1 drives diverse, cell-type-specific outputs across physiology and disease, while revealing broader principles by which multidomain scaffolds encode cellular behavior.

## 1. Introduction

SASH1 (SAM and SH3 domain-containing protein 1) is a 1247-amino-acid multidomain protein implicated in a broad spectrum of biological processes, including tumor suppression [1], innate immunity [2], migration [3], apoptosis [4], pigmentation [5], actin cytoskeleton dynamics [1], Eph receptor signaling [6], and melanocyte stem-cell maintenance [5]. Since its initial identification as a candidate tumor suppressor [7], SASH1 has been associated with inherited pigmentation disorders [8], atherosclerosis [9], Alzheimer’s disease [10], diabetic nephropathy [11], pre-eclampsia [12], and multiple cancers, including breast [13], lung [14], colorectal [3,15], gastric [16], glioma [17], melanoma [18], osteosarcoma [19], and squamous cell carcinoma [20]. Despite these diverse biological and clinical associations, a unifying mechanistic framework for SASH1 function has remained elusive.

**Figure 1:**
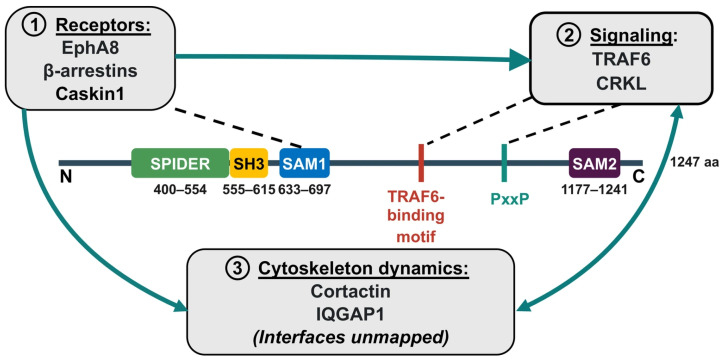
SASH1 as a context-dependent scaffold connecting receptors, signaling, and the cytoskeleton. SASH1 is proposed to organize experimentally validated binding partners into three functional modules: receptor regulation, intracellular signaling, and cytoskeletal regulation. The interactions shown are supported by varying levels of experimental evidence (see Section 4.1 for details). Several partners are themselves scaffolds or adaptor proteins, allowing SASH1 to integrate into larger signaling networks. The model is conceptual and does not imply simultaneous assembly of all partners within a single SASH1 complex. Through context-dependent partner recruitment, SASH1 may regulate diverse functions, including immune responses, tumor migration and metastasis, and pigmentation.

Recent structural and interaction data suggest that these apparently disparate functions may arise from a shared underlying role in organizing signaling architecture. SASH1 contains multiple structured domains and intrinsically disordered regions that together form a modular interaction platform capable of recruiting a diverse set of binding partners, including EphA8 [6], Caskin1 [21], β-arrestin 1 [22], TRAF6 (TNF receptor-associated factor 6) [2], CRKL [3], IQGAP1 [23], and cortactin [1], among many others. These interactions can be conceptually organized into three major functional modules (Figure 1): receptor regulation, exemplified by EphA8 and β-arrestin 1; intracellular signaling, represented by TRAF6 and CRKL; and cytoskeletal dynamics, mediated in part through IQGAP1 and cortactin. Notably, many of these binding partners are themselves central organizers of signaling or cytoskeletal architecture, supporting a model in which SASH1 functions not merely as a passive scaffold but as a higher-order coordinator of signaling networks.

Here, we propose a unified model in which SASH1 acts as a context-dependent scaffold (Figure 1). In this framework, SASH1 uses separate interaction surfaces to assemble distinct protein complexes in different cellular environments, thereby coordinating receptor regulation, intracellular signaling, and cytoskeletal remodeling in a context-dependent manner. By linking cell-surface receptor inputs to downstream signaling pathways and dynamic changes in cell architecture, SASH1 provides a mechanistic bridge between extracellular cues and cellular behavior. This organizational model explains how a single non-catalytic protein can generate diverse biological outputs across pigmentation, cancer, inflammation, and vascular biology.

In this review, we summarize the biological functions and disease associations of SASH1, examine its molecular architecture and experimentally validated interaction networks, and integrate these findings into a structure-based model of SASH1 function. We argue that SASH1’s principal role is to organize a signaling architecture that mechanistically couples receptor regulation to intracellular signaling and cytoskeletal dynamics through five structural features (see Section 4 below for details). This perspective provides a unifying framework for understanding both normal SASH1 biology and the molecular basis of SASH1-associated disease.

Beyond SASH1, this review addresses how non-catalytic scaffold proteins generate diverse biological outputs across tissues and disease states. As disease-associated variants increasingly map to multidomain scaffolds, understanding how modular interaction networks encode cellular behavior is a central challenge in cell biology. SASH1 provides a powerful model in which human genetics, structural insights, and cell biology can be integrated to link molecular architecture directly to physiological function.

## 2. SASH1 Across Cell Types and Diseases: A Common Role in Signaling and Cytoskeletal Regulation

Unlike its largely hematopoietic SLy (SH3-domain protein expressed in lymphocytes)-family relatives, SASH1 is broadly expressed across diverse tissues and cell types (Table 1), where it functions as a multifunctional intracellular adaptor and organizing scaffold [7,24,25,26,27]. Although its reported biological roles are diverse, many can be unified by a common principle: SASH1 acts as a context-dependent organizer of signaling architecture, linking receptor regulation to downstream signaling pathways and cytoskeletal remodeling. These recurring functions support our unified model and can be understood within three interrelated modules: receptor regulation, intracellular signaling, and cytoskeletal dynamics, which together help explain the broad involvement of SASH1 in physiology and disease.

The functional importance of SASH1 is underscored by loss-of-function studies: In mice, genetic ablation of *Sash1* via a gene-trap allele expressing a LacZ-tagged truncated SASH1 protein (containing the first 14 exons) leads to severe defects in lung maturation, respiratory distress, and perinatal death, demonstrating an essential role for SASH1 in endothelial signaling that supports alveolar epithelial cell maturation [22]. In humans, multiple germline SASH1 variants have been identified in more than 20 independent families with inherited pigmentation disorders [5,8,23,28,29,30,31,32,33,34]. In cultured cells, RNA interference-mediated SASH1 knockdown promotes migration, invasion, and epithelial–mesenchymal transition [3], whereas restoring SASH1 expression restrains these behaviors. Collectively, these findings demonstrate that SASH1 is a biologically important regulator whose loss or alteration produces significant developmental and cellular consequences.

### 2.1. SASH1 Functions Across Diverse Cell Types Through Recurrent Signaling and Cytoskeletal Modules

In endothelial cells, SASH1 prominently links receptor-proximal signaling to inflammatory and vascular responses. Following lipopolysaccharide stimulation, SASH1 associates with TRAF6 and IKKβ (IκB kinase β) to promote Toll-like receptor 4 (TLR4)-dependent activation of nuclear factor-κB (NF-κB) [2]. Independently, a SAM1-mediated interaction with β-arrestin 1 couples extracellular signals to Akt–endothelial nitric oxide synthase (eNOS) signaling required for lung alveolar development [22]. SASH1 also suppresses endothelial migration and angiogenic activity, highlighting a broader role in vascular homeostasis [35].

In epithelial and cancer cells, SASH1 is most strongly associated with cytoskeletal remodeling and the control of motile behavior. It localizes to F-actin-rich lamellipodia, interacts with the actin regulator cortactin, and influences membrane protrusion dynamics [1]. Consistent with these functions, SASH1 acts as a tumor suppressor in multiple cancer types [7] by restraining invasion, epithelial–mesenchymal transition (EMT), and metastasis [1,3]. Mechanistically, these effects involve interactions with signaling adaptors, such as CRKL [3], and the suppression of pro-survival pathways, including phosphoinositide 3-kinase (PI3K)/Akt signaling [13,20].

In melanocytes, SASH1 integrates signaling pathways that regulate pigmentation, cell adhesion, and stem-cell maintenance. Disease-associated SASH1 variants alter pigmentation and melanocyte migration through mechanisms involving p53–POMC–α-MSH signaling (POMC, pro-opiomelanocortin; α-MSH, α-melanocyte-stimulating hormone) [36], a Gαs–SASH1–IQGAP1–E-cadherin pathway [23], and TGF-β1 suppression [37]. More recently, SASH1 was shown to interact with tankyrase-2 to support melanocyte stem-cell maintenance, extending its role to long-term tissue homeostasis. Consistent with this function, silencing SASH1 in human primary melanocytes alters the proliferative and mitotic transcriptional programs that sustain this stem-like state [5].

In the nervous system, SASH1 appears to regulate context-dependent injury responses and glial reactivity. SASH1 expression increases during astrocyte differentiation and reactive gliosis. Experimental depletion of SASH1 reduces reactive astrocytosis while enhancing axonal regeneration and functional recovery following spinal cord injury, suggesting a role in regulating injury-induced cellular responses within the central nervous system [38]. Although the underlying molecular mechanisms remain less well defined than in other cell types and rest on a limited number of studies, these findings are consistent with a broader role for SASH1 in coordinating signal-dependent responses to environmental change.

**Table 1:** SASH1 functions across cell types.

Cell Type	Key Functions	Signaling Pathways
Endothelial cells	Scaffolds TLR4–NF-κB inflammatory signaling [2]; couples extracellular cues to Akt–eNOS–nitric oxide for alveolar/lung maturation [22]; restrains endothelial migration and angiogenesis [35]	TLR4–TRAF6–TAK1/IKK–NF-κB; β-arrestin 1–eNOS
Epithelial/cancer cells	Acts as a tumor suppressor, restrains invasion, EMT and metastasis [1,3]; promotes apoptosis [4]	CRKL–SRC [3]; PI3K–Akt [20]; caspase-3 cleavage → nuclear NF-κB [4]
Melanocytes	Promotes stemness of the melanocyte lineage; regulates melanin synthesis and melanocyte migration	tankyrase-2 [5]; p53–POMC–α-MSH [36]; Gαs–IQGAP1–E-cadherin [23]; TGF-β1 suppression [37]
Astrocytes/neural cells	Induction during astrocyte differentiation and reactive gliosis; depletion enhances axonal regeneration and recovery after spinal-cord injury; anchors cytoplasmic PKM2 after traumatic brain injury	Reactive astrocytosis [38]; PKM2 anchoring [39]
Cytoskeletal (general)	Localizes to F-actin-rich lamellipodia and membrane ruffles; binds cortactin; organizes actin remodeling, cell adhesion and migration	Cortactin–F-actin [1]; CRKL [3]; IQGAP1 [23]

EMT, epithelial–mesenchymal transition; eNOS, endothelial nitric oxide synthase; NF-κB, nuclear factor-κB; PKM2, pyruvate kinase M2; TAK1, TGF-β-activated kinase 1; TLR4, Toll-like receptor 4

### 2.2. Dysregulated SASH1 Is Associated with a Broad Spectrum of Human Diseases

These cell-type-specific functions translate into an equally broad disease spectrum (Table 2). Germline SASH1 variants cause inherited pigmentation disorders, including autosomal dominant dyschromatosis universalis hereditaria, lentiginous phenotypes [8,23,28], and premature hair graying [5], as well as a recessive syndromic genodermatosis with hyper- and hypopigmentation, palmoplantar keratoderma, and skin carcinoma [29].

Beyond these Mendelian disorders, altered SASH1 expression or genetic variation has been associated with vascular and complex diseases. SASH1 is elevated in atherosclerotic arteries from smokers and has been proposed as a molecular link between cigarette smoking and atherosclerosis [9]. Genetic, epidemiological, and expression studies have further implicated SASH1 in conditions including Alzheimer’s disease [10], diabetic nephropathy [11], and pre-eclampsia [12]. Unlike inherited pigmentation disorders, where germline SASH1 variants provide direct genetic evidence of causation, associations with complex diseases are supported primarily by expression, functional, or epidemiological studies and remain less firmly established mechanistically.

Reduced SASH1 expression, or loss of its tumor-suppressor function, has also been reported in numerous solid tumors, including breast, colorectal, lung, gastric, glioma, melanoma, and osteosarcoma, where it generally correlates with greater invasion, metastasis, and poorer clinical outcomes [3,7,13,14,15,16,17,18,19]. Conversely, restoration of SASH1 expression, including pharmacologic upregulation by the antihistamine chloropyramine, suppresses malignant phenotypes in breast cancer cells [40].

Collectively, these observations reveal a striking functional versatility: a single protein participates in inflammatory signaling, vascular function, cytoskeletal remodeling, pigmentation, stem-cell maintenance, tumor suppression, and neural injury responses. Resolving this apparent functional diversity requires moving beyond individual cell types and disease contexts to examine the multidomain molecular architecture that enables these activities, which is the focus of the following sections.

## 3. Biochemical Properties, Subcellular Localization, and Molecular Architecture of SASH1

### 3.1. Biochemical Properties and Subcellular Localization of SASH1: Dynamic Partitioning Across Signaling Compartments

SASH1 is an intracellular scaffold protein rather than a membrane protein. Consistent with its proposed role as a signaling organizer, SASH1 exhibits dynamic subcellular localization, partitioning between the cytoplasm and nucleus while transiently associating with membrane-proximal signaling complexes and the cortical actin cytoskeleton. Current evidence indicates that its localization is highly context-dependent and changes according to cell type and signaling state.

The first detailed characterization of endogenous SASH1 demonstrated that it localizes to both the nucleus and cytoplasm in epithelial cells, with enrichment at lamellipodia, membrane ruffles, and cell–cell contact regions, where it co-localizes with filamentous actin and cortactin [1]. Biochemical fractionation confirmed the presence of endogenous SASH1 in cytosolic, crude membrane, and nuclear fractions, indicating reversible association with membrane-associated signaling and cytoskeletal complexes rather than stable membrane insertion [1]. Consistent with these observations, SASH1 expression promotes actin polymerization, associates with cortactin, and regulates cell adhesion and migration [1].

Subsequent studies further demonstrated that SASH1 can be recruited to membrane-proximal signaling complexes. In FADD-deficient mouse embryonic fibroblasts, proteomic analysis identified SASH1 within detergent-resistant membrane (lipid raft) fractions following TLR4 activation, where it functions as a scaffold linking TRAF6, TAK1, and IKK to promote NF-κB signaling [2]. These findings indicate that membrane association is dynamic and reflects recruitment into receptor-associated signaling complexes rather than intrinsic membrane localization.

SASH1 localization is also regulated during apoptosis. Under basal conditions, full-length SASH1 is predominantly cytoplasmic; however, activation of caspase-3 cleaves SASH1 at Asp230, generating a C-terminal fragment that translocates to the nucleus and associates with chromatin [4]. This cleavage-dependent relocalization is required for efficient NF-κB activation and the promotion of apoptosis, illustrating that SASH1 can transition from a cytoplasmic scaffold to a nuclear signaling regulator in response to cellular stress [4].

Collectively, these observations support a model in which SASH1 is a dynamic intracellular scaffold that is neither constitutively cytoplasmic nor exclusively nuclear. Instead, SASH1 partitions between soluble cytoplasmic and nuclear pools while being transiently recruited to membrane-proximal signaling complexes and cortical actin structures. This dynamic localization is consistent with the central hypothesis developed in this review: SASH1 functions as a context-dependent scaffold that links receptor activation at the plasma membrane to intracellular signaling pathways, cytoskeletal remodeling, and, under specific conditions, nuclear responses.

Two putative nuclear localization signals (NLS1 and NLS2) were initially identified by sequence analysis and deletion mapping, which implicated the N-terminal region of SASH1 in nuclear targeting [1]. Martini et al. showed that endogenous SASH1 is distributed between the nucleus and cytoplasm and demonstrated that deletion of the C-terminal portion of SASH1, while retaining both predicted NLSs, resulted in marked nuclear accumulation, whereas deletion of the N-terminal region containing the predicted NLSs largely abolished nuclear enrichment [1]. These findings suggest that functional nuclear localization determinants reside within the N-terminal portion of the protein.

Subsequent work provided direct experimental support for NLS function [3]. Franke et al. showed that site-directed mutagenesis of NLS1 and NLS2, individually or in combination, impaired nuclear localization of an N-terminal SASH1 construct, demonstrating that both motifs contribute to nuclear import. In the same study, full-length SASH1 was observed predominantly in the cytoplasm and at focal-adhesion-like structures, but a nuclear pool was also detected, indicating that SASH1 undergoes nucleocytoplasmic shuttling.

Further evidence for regulated nuclear trafficking was provided by Burgess et al., who demonstrated that caspase-3-mediated cleavage during apoptosis generates a C-terminal SASH1 fragment that translocates from the cytoplasm to the nucleus [4]. Notably, this fragment retains both NLSs, consistent with a potential role for these motifs in nuclear import.

Collectively, these observations support the existence of functional nuclear import determinants within SASH1 and indicate that its nuclear localization is dynamically regulated.

### 3.2. Domain Organization

**Figure 2:**
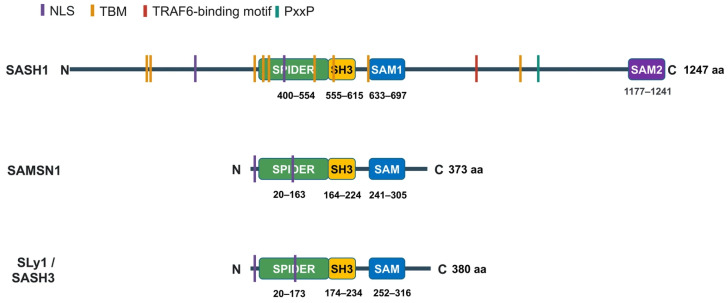
SASH1 domain architecture: conserved core with unique C-terminal extensions. Linear domain maps (to scale) of human SASH1 (1247 aa; UniProt O94885) and its SLy-family paralogs SAMSN1 (373 aa) and SLY1/SASH3 (380 aa). All three proteins share a conserved central module comprising an intrinsically disordered SPIDER (SASH1 residues ~400–554), followed by an SH3 domain (555–615) and a SAM1 domain (633–697). SASH1 is uniquely distinguished by both an extended N-terminal undefined region and a long proline-rich linker, as well as a unique C-terminal second SAM domain (SAM2; 1177–1241). Annotated motifs include two nuclear localization signals (NLS), nine predicted tankyrase-binding motifs (TBMs; four within the SPIDER), a TRAF6-binding motif (852–860), and a CRKL-binding PxxP motif (985–990).

SASH1 is a 1247-amino-acid multidomain scaffold protein (UniProt: O94885) with no known catalytic activity [1,4]. SASH1 belongs to the SLy family of adaptor/scaffold proteins, which includes SAMSN1 and SLY1/SASH3, and is distinguished from its paralogs by the presence of a second C-terminal SAM domain and broader tissue expression [27] (Figure 2). Its architecture is dominated by interaction modules, consistent with a role in organizing signaling complexes. From the N- to the C-terminus, SASH1 comprises the intrinsically disordered SPIDER (residues 400–554) [41], an SH3 domain (residues 555–615), the SAM1 domain (residues 633–697), an extended proline-rich linker (~480 residues), and a second SAM domain (SAM2; residues 1177–1241) [42].

Several predicted and experimentally validated functional motifs are embedded within this organization. These include nine predicted tankyrase-binding motifs (TBMs; four experimentally validated) [5,43], two validated nuclear localization signals (NLSs), a validated TRAF6-binding motif (residues 852–860; DVPTEVTEP) [2], a validated proline-rich PxxP motif critical for CRKL binding (residues 985–990) (Figure 2), and multiple predicted phosphorylation sites.

Among these features, the central SPIDER–SH3–SAM1 region contains the highest density of characterized interaction motifs. Four canonical TBMs located within SPIDER, including the disease-associated TBM6 and TBM7 motifs, bind the ankyrin-repeat cluster 4 (ARC4) domain of tankyrase-2 (TNKS2), as demonstrated by nuclear magnetic resonance (NMR) and biochemical studies [5,43].

SASH1 belongs to the relatively small family of multi-SAM-containing proteins (MSCPs), which harbor two or more SAM domains within a single polypeptide chain [42]. Compared with other MSCPs such as Caskin1, Liprin, and ANKS1B, SASH1 is distinguished by the unusually large separation between SAM1 and SAM2, which are spaced nearly 500 residues apart [42]. Whether these two SAM domains interact directly remains unknown. In several MSCPs, adjacent domains are organized into supra-domains that act as a single scaffolding unit [42]. In SASH1, the close sequence spacing of SPIDER, SH3, and SAM1, which together harbor most characterized interaction surfaces and nearly all reported pigmentation-associated variants, raises the possibility of a comparable arrangement, although this remains to be tested experimentally.

### 3.3. Evolutionary Conservation

SASH1 is conserved across vertebrates, with the strongest sequence conservation concentrated within the SPIDER–SH3–SAM1 core region [5]. The SLy family, consisting of SASH1, SLY1/SASH3, and SAMSN1, shares this conserved central architecture [27] (Figure 2).

Across vertebrate SASH1 orthologs, SPIDER is highly conserved, consistent with its functional importance in TBM-dependent interactions [5]. The key acidic residues in SAM1 (E660, D663, E669, and D672) that mediate EphA8 binding are also conserved across vertebrates [6]. Functionally, the aromatic Y659 and the acidic E660 and D663 are each individually essential (alanine substitution of any of these abolishes binding), while E669 and D672 contribute further; on the receptor side, cancer-associated EphA8 mutations at this interface disrupt the interaction [6]. The conservation of these motifs suggests strong evolutionary pressure to maintain their structural and interaction properties.

Comparison with other SLy family members reveals a notable distinction. Whereas all family members contain the central SPIDER–SH3–SAM1 region, only SASH1 possesses a well-conserved second SAM domain (SAM2) at the C-terminus. SAM2 is completely absent in SLY1/SASH3 and SAMSN1 [27], indicating that it may represent a unique structural and functional feature of SASH1.

### 3.4. Predicted Overall Structure of SASH1 by AlphaFold2

**Figure 3:**
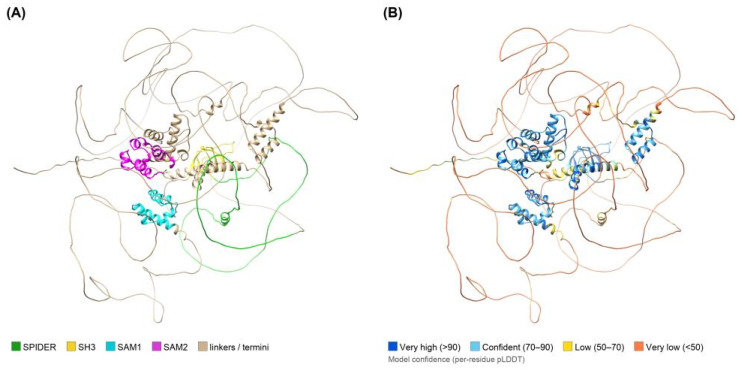
Predicted structure of human SASH1 (UniProt O94885; AlphaFold2 AF-O94885-F1), colored by domain in (**A**) and by per-residue model confidence (predicted Local Distance Difference Test, pLDDT) in (**B**). (**A**) The SH3, SAM1, and SAM2 domains are predicted to form the principal structured regions of SASH1. In contrast, the SPIDER is largely unstructured, consistent with NMR evidence that it is intrinsically disordered. The SPIDER–SH3–SAM1 module is predicted to form a relatively compact central core, whereas both the extended N-terminal region and the long C-terminal linker are predicted to be flexible and low-confidence. A SAM-like helical bundle (~1092–1170) is predicted adjacent to SAM2 (this region falls within the linker/terminus coloring in panel (**A**) but is resolved as a high-confidence prediction in panel (**B**)), suggesting a supra-domain arrangement similar to those observed in other multi-SAM proteins. As a predictive AlphaFold2 model, confidence decreases outside well-folded domains, and inter-domain contacts remain uncertain in solution; experimental multidomain structures will be required to validate these arrangements. (**B**) The same model colored by pLDDT: the folded SH3, SAM1, and SAM2 domains are predicted with high confidence, whereas the SPIDER, the inter-domain linkers, and the extended N- and C-terminal regions are predicted with low confidence. Approximately 64% of residues score below pLDDT 50, underscoring that the predicted inter-domain arrangement is uncertain and requires experimental validation.

Although several regions of SASH1 have been characterized experimentally, no global structure of the full-length protein has been reported. Consequently, AlphaFold2 currently provides the most complete model of SASH1 structural features (Ref. [44]; UniProt: O94885) (Figure 3).

The AlphaFold2 model is broadly consistent with available experimental data. The SH3 and SAM2 domains are predicted with high confidence and closely match their experimentally determined NMR structures (Protein Data Bank [PDB] 2EBP and 2DL0, respectively). The model also supports the experimentally observed distinction between ordered and disordered regions within SASH1, predicting SPIDER to be largely unstructured while the SH3 and SAM2 domains adopt compact globular folds. These predictions agree well with NMR studies demonstrating that SPIDER is intrinsically disordered and that SH3 and SAM2 are independently folded domains [41,42].

Beyond validating known features, AlphaFold2 provides insight into the overall organization of the protein. In the model, SPIDER, SH3, and SAM1 are positioned within a relatively compact central region; however, because the connecting linkers are predicted with low confidence, this compact arrangement should be regarded as a hypothesis rather than an established feature of the full-length protein. The long C-terminal linker between SAM1 and SAM2 is likewise predicted to be largely extended and of lower confidence, consistent with substantial flexibility. While not definitive, the model predicts a partially structured SH3–SAM1 linker that may contribute to local domain organization.

A particularly intriguing feature is the prediction of a SAM-like helical bundle spanning residues 1092–1170 immediately N-terminal to SAM2. This region is predicted with relatively high confidence. It is predicted to pack against SAM2 in an arrangement that would resemble the supra-domain architectures observed in other MSCPs such as Liprin and Caskin family members [42]. Because this putative domain remains experimentally uncharacterized, it could equally represent a computational artifact as a genuine domain; whether it constitutes a bona fide folded domain and contributes to intra- or intermolecular interactions therefore remains an open question for future studies.

Despite these insights, caution is warranted when interpreting the AlphaFold2 model. Outside the folded domains, confidence decreases substantially because there are few to no predicted intramolecular interactions to calculate structural folds, and the model does not establish whether predicted domain contacts occur in solution. Experimental determination of multidomain and full-length SASH1 structures will therefore be required to validate the proposed architecture and define the conformational landscape of the intact protein.

### 3.5. Structural Properties of Individual Domains

The location, structure, and function of each region and domain of SASH1 are summarized in Table 3 and described below in detail.

**SPIDER.** The SLy Proteins Associated Disordered Region (SPIDER; residues ~400–554) is intrinsically disordered, as demonstrated by NMR [41]. Despite its lack of stable secondary structure, SPIDER contains a high density of functional motifs, including at least one of the two NLSs (NLS2, residues 452–468) [27] and multiple TBMs [5,43]. SASH1 can localize to the nucleus [1], consistent with a functional nuclear import signal in this region. NMR studies demonstrated that SPIDER contains four functional TBMs capable of binding the ARC4 domain of TNKS2 [5,43]. As observed for other tankyrase interactors, including RNF146, these motifs can mediate multivalent interactions with tankyrase [46].

**Figure 4:**
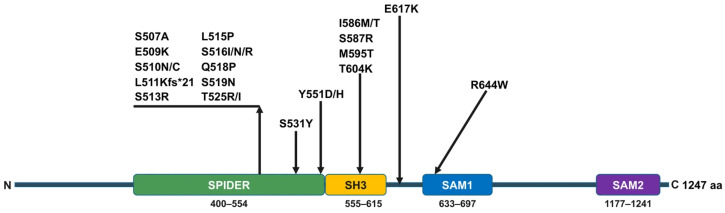
Reported pigmentation-associated human SASH1 variants (UniProt O94885) mapped onto the domain backbone with all annotated motifs. More than 70% of variants localize to the SPIDER, with strong clustering in and around TBM6 (512–519) and TBM7 (552–559), two of the four SPIDER tankyrase-binding motifs that contribute to the multivalent SASH1–tankyrase-2 interaction interface. Approximately 20% map to the SH3 domain, notably on the surface opposite the canonical ligand-binding site. Only rare variants occur within SAM1 (e.g., R644W), and apart from E617K immediately C-terminal to the SH3 domain, no variants have been reported elsewhere in SASH1.

This arrangement may allow a single SPIDER to engage multiple TNKS2 ankyrin-repeat clusters simultaneously or dynamically exchange among binding sites, a characteristic feature of multivalent interactions mediated by intrinsically disordered proteins [46]. The binding affinity of TBMs is known to be influenced by local sequence context, including neighboring motifs and the identity of residues flanking the TBM consensus sequence, each of which shapes the local backbone conformation of the motif [47,48,49]. More than 70% of currently reported pigmentation-associated variants are located within SPIDER, particularly near TBM6 (residues 512–519) and TBM7 (residues 552–559), highlighting the functional importance of this region [5] (Figure 4).

**Figure 5:**
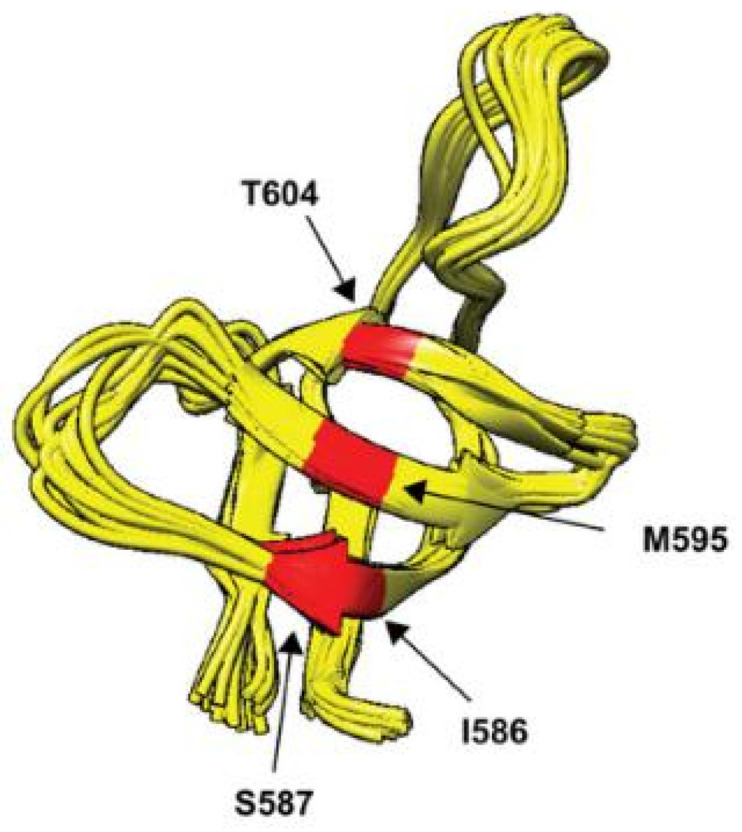
SASH1 SH3 domain structure and variant mapping visualized in UCSF Chimera. NMR ensemble of the SASH1 SH3 domain (residues 555–615; PDB 2EBP), which adopts a canonical SH3 fold consisting of a compact β-barrel at physiological pH. Pigmentation-associated variant positions (I586, S587, M595, and T604) are highlighted in red and cluster on the face opposite the canonical ligand-binding surface. This distribution suggests that these variants primarily affect overall domain stability or contribute to higher-order organizational roles within the SPIDER–SH3–SAM1 module, rather than disrupting classical proline-rich ligand recognition.

**SH3 domain.** The SH3 domain (residues 555–615; PDB: 2EBP) adopts a canonical SH3-domain fold consisting of a compact β-barrel at physiological pH (Figure 5, generated by UCSF Chimera [50]). Together with adjacent sequences, this region contributes to targeting SASH1 to F-actin-rich lamellipodia and membrane ruffles, where it colocalizes with cortactin and the actin cytoskeleton [1]. Approximately 20% of reported pigmentation-associated variants map to this domain (Figure 4), notably on the surface opposite the canonical SH3 ligand-binding interface. This distribution suggests that these variants may perturb global domain stability or contribute to higher-order macromolecular functions, such as regulating or stabilizing the adjacent SAM1 domain. Positioned between the intrinsically disordered SPIDER and the structurally dynamic SAM1, the SH3 domain is well placed to help organize the central architecture of SASH1. Although SH3 domains typically recognize proline-rich ligands, the binding partners and structural determinants governing ligand recognition by the isolated SASH1 SH3 domain remain incompletely defined.

**Figure 6:**
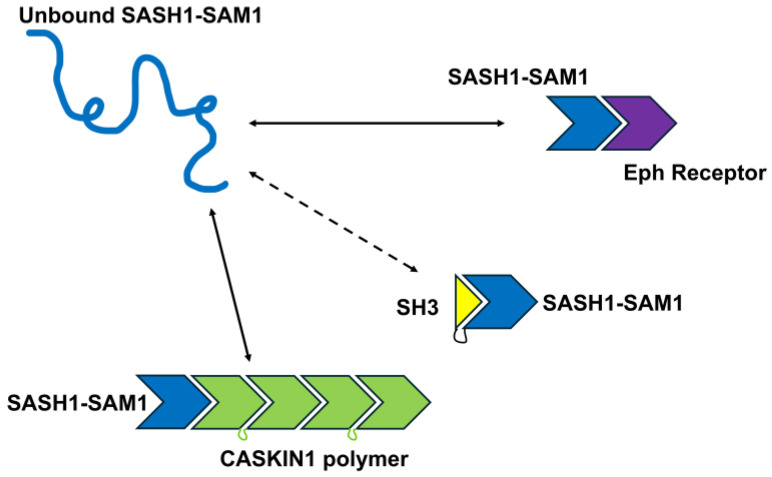
SASH1 SAM1: a conformationally regulated SAM domain stabilized by partner binding. Unlike canonical SAM domains, the isolated SAM1 domain (residues 633–697) of SASH1 is structurally heterogeneous, predominantly disordered, with a minor folded population. Upon binding to the EphA8 SAM domain, SAM1 adopts a canonical five-helix fold (structurally resolved for the EphA8 complex; PDB 8J1I); it also engages the Caskin1 SAM domain. Whether the adjacent SH3 domain likewise stabilizes SAM1 has been proposed but not tested directly and is therefore shown as a dashed arrow. These observations suggest that its intrinsic instability serves a regulatory function.

**SAM1.** SAM1 (residues 633–697) exhibits atypical structural behavior compared with canonical SAM domains (Figure 6). Whereas most SAM domains are well folded, isolated SAM1 is conformationally heterogeneous, existing predominantly as a disordered monomer (~98%), with a minor folded, oligomeric population [45]. This behavior contrasts with the SLy1/SASH3 SAM domain, which forms a stable homodimer [51], and is consistent with other multi-SAM-containing proteins in which at least one SAM domain is structurally unstable [42] and proposed to have a regulatory function [52].

Importantly, SAM1 adopts a stable, canonical five-helix fold upon partner engagement. It becomes structured upon binding to the EphA8 SAM domain, enabling crystallographic analysis (PDB 8J1I) [6]. It has been proposed that the adjacent SH3 domain may likewise stabilize the SAM1 fold, although this has not been tested directly [42]. The mouse SASH1–EphA8 complex reveals a conserved SAM–SAM interface involving residues corresponding to Y659, E660, D663, E669, and D672 in human SASH1, with E660 or D663 substitutions abolishing binding, establishing these residues as critical determinants of complex formation [6]. SAM1 also interacts with Caskin1, modulating its SAM-mediated polymerization [21], and mediates association with β-arrestin 1 [22]. Whether distinct binding partners engage overlapping or separate interaction surfaces and whether SAM1 folding occurs via conformational selection or induced fit remain unresolved.

**Figure 7:**
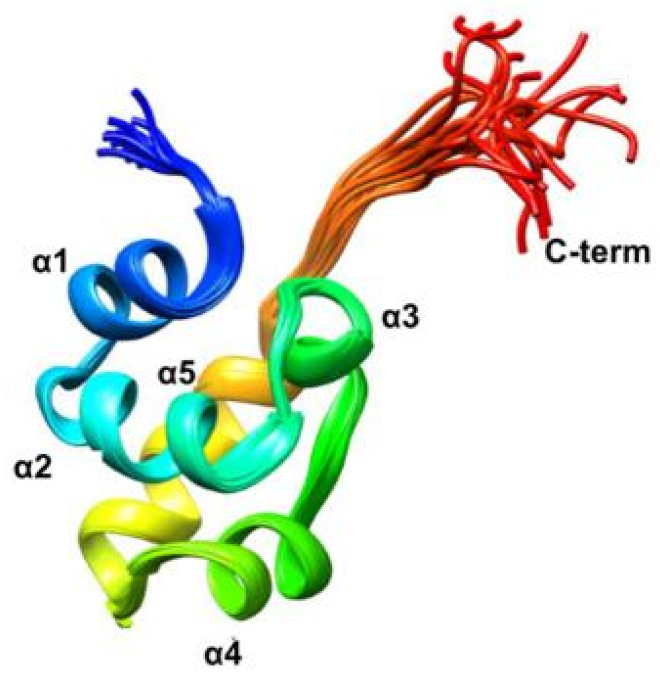
SASH1 SAM2 domain structure visualized in UCSF Chimera. NMR ensemble of the C-terminal SASH1 SAM2 domain (residues 1177–1241; PDB 2DL0), which adopts a canonical five-helix SAM fold (helices α1–α5 labeled) and is monomeric in solution. Unlike many SAM domains, SAM2 has not been observed to self-associate, and no binding partners have yet been identified. Cloning artifacts have been removed from the displayed model. AlphaFold2 additionally predicts an adjacent SAM-like helical bundle (~1092–1170) packing against SAM2 (see also Figure 3).

**SAM2.** SAM2 (residues 1177–1241; PDB: 2DL0) adopts a canonical five-helix SAM-domain fold and is monomeric in solution (Figure 7). Unlike many SAM domains, SAM2 has not been reported to self-associate [42,53], and no binding partners have yet been identified. The AlphaFold2 model predicts that SAM2 forms a structural unit with an adjacent SAM-like helical bundle located immediately N-terminal to the domain [42]. Experimental validation of this predicted architecture and identification of SAM2-associated proteins represent important priorities for future work.

Collectively, current structural studies indicate that SASH1 contains a mixture of ordered and intrinsically disordered elements organized around multiple conserved interaction modules. Although several individual domains have now been structurally characterized, the architecture and conformational dynamics of full-length SASH1 remain largely unexplored.

## 4. SASH1 as a Context-Dependent Multi-Docking Scaffold

**Figure 8:**
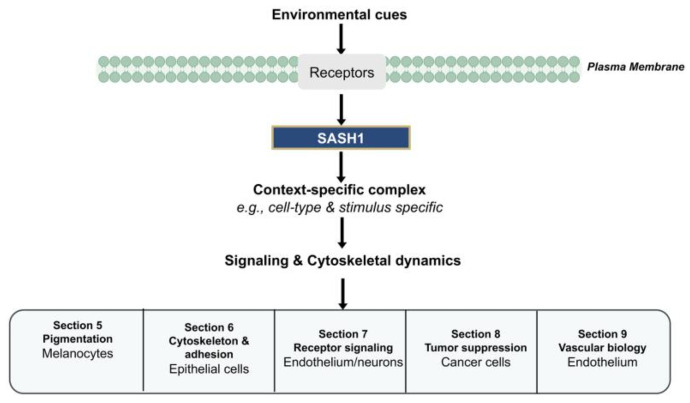
Integrative model of SASH1 function. Environmental cues sensed by cell-surface receptors are relayed to SASH1, which assembles distinct, context-specific complexes determined by cell type and stimulus rather than operating within a single fixed pathway. Through this combinatorial engagement, SASH1 links upstream inputs to diverse downstream outputs.

The functional diversity of SASH1 can be explained by a unifying model in which SASH1 functions as a context-dependent scaffold (Figure 1). Rather than serving as a dedicated component of a single signaling pathway, SASH1 acts as a molecular platform that assembles distinct protein complexes in different cellular environments (Figure 8). Its multifunctionality arises from the combination of multiple separable interaction modules, extensive intrinsic disorder, a unique dual-SAM architecture for heterotypic interactions, the ability to recruit other scaffold and adaptor proteins, and dynamic post-translational regulation. Together, these features enable SASH1 to couple diverse upstream signals to cell-type-specific responses in signaling, transcription, and cytoskeletal organization (Table 4).

### 4.1. A Modular Protein with Separable Interaction Surfaces

A key feature underlying SASH1 multifunctionality is the spatial separation of its interaction interfaces across distinct domains and linear motifs. Experimental studies have identified multiple binding surfaces that recruit different partners through independent mechanisms (Table 5). The intrinsically disordered SPIDER contains multiple tankyrase-binding motifs that mediate interaction with TNKS2 and connect SASH1 to tankyrase-dependent regulation of stem-like characteristics of the melanocyte lineage [5,43]. The SAM1 domain serves as a multifunctional interaction hub, mediating interactions with Eph receptor SAM domains [6] and β-arrestin 1 [22]. A TRAF6-binding motif links SASH1 to inflammatory signaling downstream of TLR4 [2], whereas a canonical PxxP motif engages the SH3 domain of CRKL and modulates CRKL-dependent SRC signaling [3,54]. Additional interactions with IQGAP1 and Gαs further connect SASH1 to pathways regulating melanocyte migration and melanogenesis [23,37].

These interactions differ substantially in the strength of supporting evidence: the EphA8 interaction has been resolved structurally by co-crystallography, and the tankyrase-2 interaction mapped by NMR titration and quantified by microscale thermophoresis; the CRKL and Caskin1 interactions are biochemically validated by direct in vitro binding assays (and, for CRKL, interface mutagenesis); the β-arrestin 1 interaction is supported by yeast two-hybrid and reciprocal co-immunoprecipitation, with its interface localized to SAM1 by deletion mapping; and the cortactin, IQGAP1, and PKM2 interactions are currently supported mainly by co-immunoprecipitation, with their SASH1-binding interfaces still unmapped. We therefore treat these partners as differing in the confidence with which they can be assigned to the model, rather than as equally established.

**Table 5:** Experimentally validated SASH1 interaction partners.

Partner	Category	SASH1 Element and Evidence Tier	Experimental Evidence (Method)	Functional Output	Primary References
TNKS2 (tankyrase-2)	PARP enzyme and scaffold	SPIDER (TBMs) Biochemical	Y2H; co-IP; NMR titration; MST	Melanocyte stem-cell maintenance; pigmentation	[5,43]
EphA8	Receptor tyrosine kinase	SAM1 Structural	Co-crystal (PDB 8J1I); mutagenesis	Eph–ephrin signaling; cell positioning	[6]
Caskin1	Tandem-SAM scaffold	SAM1 Biochemical	Y2H; SEC; ITC	Disassembles Caskin1 SAM polymer	[21]
β-arrestin 1 (ARRB1)	Signaling scaffold	SAM1 Cellular	Y2H; reciprocal co-IP	Akt–eNOS–nitric oxide signaling	[22]
TRAF6	E3-ligase adaptor	TRAF6-binding motif (res. 852–860) Cellular	Co-IP; knockdown	TLR4–NF-κB inflammatory signaling	[2]
TAK1, IKKα, IKKβ	Ser/Thr kinases	— Cellular	Co-IP	TLR4 signalosome assembly	[2]
CRKL	SH3/SH2 adaptor	Central PxxP (res. 985–990) Biochemical	Y2H; co-IP/MS; alanine substitution; DMR (Kd ≈ 7.4 µM)	Anti-EMT; metastasis suppression	[3]
Cortactin	Actin-nucleation scaffold	Full-length only Unmapped	co-IP (endogenous); cell fractionation; immunofluorescence; deletion mapping (no fragment sufficed)	Actin/lamellipodia organization	[1]
IQGAP1	Multidomain scaffold	— Unmapped	AP-MS; co-IP	Gαs–IQGAP1–E-cadherin; melanocyte adhesion	[23]
PKM2 (pyruvate kinase M2)	Glycolytic enzyme	— Unmapped	AP-MS (astrocytes); co-IP	Cytoplasmic PKM2 anchoring (astrocytes, TBI)	[39]

AP-MS, affinity purification–mass spectrometry; ARRB1, β-arrestin 1; co-IP, co-immunoprecipitation; DMR, dynamic mass redistribution; ITC, isothermal titration calorimetry; MST, microscale thermophoresis; PARP, poly(ADP-ribose) polymerase; SEC, size-exclusion chromatography; SH2, SRC-homology-2 domain; TBI, traumatic brain injury; Y2H, yeast two-hybrid. Evidence tier: Structural, three-dimensional structure determined; Biochemical, direct in vitro binding or interface mapping; Cellular, co-immunoprecipitation and/or yeast two-hybrid association; Unmapped, interaction detected but the SASH1-binding interface not localized.

Throughout the discussion that follows, readers should refer to the “SASH1 element and evidence tier” column in Table 5, which summarizes the type of evidence supporting each interaction. Because the available evidence ranges from structural and biochemical validation to functional cellular studies, these classifications provide important context for assessing the strength and limitations of the current data.

Because these interaction surfaces are in distinct regions of the protein, SASH1 has the potential to engage different binding partners sequentially and, in principle, simultaneously. However, simultaneous recruitment of multiple partners by full-length SASH1 has not yet been demonstrated and remains an important open question (Section 11). This modular organization enables SASH1 to function as a scaffold for the assembly of signaling complexes that vary according to cellular context and/or external stimuli.

### 4.2. Intrinsic Disorder Enables Interaction Plasticity

Two of the most functionally important regions of SASH1—the SPIDER and SAM1 domains—exhibit substantial structural flexibility. The SPIDER is intrinsically disordered and contains multiple short linear motifs involved in protein binding, as well as disease-associated mutations [41]. In contrast to most SAM domains, which form stable helical bundles and frequently self-associate, SASH1 SAM1 displays pronounced conformational heterogeneity and limited self-polymerization [45].

The disordered nature of some SASH1 domains could confer unique features for multivalent binding and post-translational modifications (PTMs). These two features, separately or combined, permit the recruitment of diverse proteins in space and time. The disordered regions could expose multiple short linear motifs [55] that mediate specific interactions. Additionally, despite their disordered nature, these regions can adopt secondary-structure propensities that form molecular recognition motifs [56]. Such flexibility may allow SASH1 to engage a diverse set of partners, including tankyrases, Eph receptors, β-arrestins, and potentially other SAM-containing proteins.

Additionally, the disordered nature could increase SASH1’s functional states through PTMs [57]. The concentration of disease-associated variants within the SPIDER further highlights the functional importance of this disordered interaction platform.

The extensive intrinsic disorder and multivalent, motif-based interactions that characterize SASH1 are also hallmarks of proteins that form biomolecular condensates through liquid–liquid phase separation (LLPS). Scaffold and signaling proteins that combine disordered regions with multiple short linear motifs can concentrate binding partners into membraneless compartments, locally increasing the efficiency of assembly and signaling [58,59]. In reconstituted systems, multivalent domain–motif interactions of this type are sufficient to drive phase separation of signaling scaffolds and to couple receptor input to actin assembly [60]. Whether SASH1 undergoes LLPS is currently unknown—no condensate behavior has been directly demonstrated—but its disordered SPIDER, multivalent tankyrase-binding motifs, and enrichment at discrete structures such as F-actin-rich lamellipodia make phase separation a plausible and testable hypothesis. Live-cell imaging of puncta dynamics, sensitivity to 1,6-hexanediol, and fluorescence recovery after photobleaching could determine whether SASH1 organizes signaling in part through condensate formation.

### 4.3. Dual SAM Domains Specialize SASH1 for Heterotypic Interactions

SASH1 is unique among SLy family proteins in possessing two SAM domains [27]. Available structural and biochemical evidence suggests that these domains are optimized for selective heterotypic interactions rather than extensive homopolymerization. SAM1 interacts with Eph receptor SAM domains through a conserved mid-loop/end-helix (ML/EH) binding interface [6] and may also participate in interactions with other SAM-containing proteins.

This architecture potentially enables SASH1 to integrate directly into receptor-associated signaling complexes without possessing intrinsic catalytic activity. By functioning as a SAM-mediated adaptor, SASH1 may regulate access of downstream signaling proteins to receptor complexes and facilitate communication between otherwise independent signaling pathways. Whether the two SAM domains function in a coordinated manner or engage distinct sets of binding partners remains an important unanswered question.

### 4.4. Post-Translational Regulation as a Signaling Switchboard

Multiple post-translational mechanisms likely regulate the composition and function of SASH1-containing complexes. In TLR4-stimulated cells, SASH1 promotes K63-linked polyubiquitination of TRAF6 and facilitates assembly of the TRAF6–TAK1–IKK signaling axis [2]. β-Arrestins, which also interact with SASH1, provide potential feedback regulation through a parallel, TRAF6-independent branch of this network [22]. Caspase-mediated cleavage can relocalize SASH1 from the cytoplasm to the nucleus, converting a cytoplasmic scaffold into a regulator of transcriptional responses [4]. Additional phosphorylation-dependent mechanisms may regulate subcellular localization and the selection of interaction partners. For example, SASH1 is itself a phosphorylation-regulated 14-3-3-binding protein: insulin-like growth factor 1 (IGF-1) and phorbol 12-myristate 13-acetate (PMA) signaling promote phosphorylation of Ser90 within a consensus 14-3-3 recognition motif, generating a regulated docking site for 14-3-3 proteins [61].

These regulatory processes suggest that SASH1 functions not as a static scaffold, but as a dynamically regulated signaling platform whose interaction network can be remodeled in response to cellular stimuli.

### 4.5. SASH1 as a Scaffold-of-Scaffolds

A distinctive feature of SASH1 is that several of its experimentally validated binding partners are themselves scaffold or adaptor proteins that assemble multi-protein complexes by linking two or more binding partners (Table 5). This observation suggests that SASH1 functions not merely as a conventional scaffold that recruits individual signaling proteins but as a higher-order organizer that connects pre-existing signaling modules. In this hierarchical model, SASH1 links upstream receptors and signaling inputs to networks already structured by other scaffold or adaptor proteins. By engaging proteins such as IQGAP1, tankyrase-2, β-arrestin 1, cortactin, and CRKL, SASH1 coordinates entire signaling modules rather than isolated downstream effectors [62,63].

A representative example is IQGAP1, which associates with SASH1 in melanocytes [23]. IQGAP1 is a multifunctional scaffold that coordinates receptor signaling, small GTPases, cell-adhesion complexes, and actin cytoskeletal dynamics through interactions with numerous binding partners [64,65,66,67]. Consequently, recruitment of IQGAP1 potentially links SASH1 to an extensive signaling and cytoskeletal network. A similar organizational principle is evident in several other validated SASH1 interactions. Through SPIDER, SASH1 binds tankyrase-2, a scaffold-like poly(ADP-ribose) polymerase (PARP) that organizes protein complexes involved in Wnt and telomere regulation [68,69,70]. Through SAM1, SASH1 interacts with β-arrestin 1, a multifunctional adaptor that assembles receptor-associated signaling complexes and regulates cytoskeletal remodeling [71,72,73,74,75]. SASH1 also associates with cortactin, an actin-organizing scaffold that coordinates Arp2/3-dependent actin assembly [1,76,77]. In addition, SASH1 directly binds the adaptor protein CRKL through a canonical SH3-binding motif [3]. Because CRKL serves as a major signaling hub downstream of receptor tyrosine kinases and integrins [78,79], this interaction further supports the notion that SASH1 preferentially engages proteins with network-organizing functions.

Collectively, these interactions support a hierarchical model in which SASH1 links upstream receptors and signaling inputs to networks already organized by other scaffold proteins. By engaging scaffold and adaptor proteins such as IQGAP1, tankyrase-2, β-arrestins, cortactin, and CRKL, SASH1 has the potential to coordinate entire signaling modules [62,63] rather than individual downstream effectors. However, this hierarchical ‘scaffold-of-scaffolds’ organization remains a working model, as direct evidence that full-length SASH1 simultaneously assembles multiple pre-existing scaffold complexes is not yet available.

### 4.6. A Unified Model for SASH1 Multifunctionality

The diverse biological functions of SASH1 can be reconciled through a common mechanistic framework: rather than functioning within a single signaling pathway, SASH1 acts as a context-dependent signaling organizer that assembles distinct protein complexes in different cellular environments. A recurring theme across the current literature is that SASH1 operates at the interface between receptor signaling and cytoskeletal remodeling. Upstream, SASH1 participates in receptor-associated signaling pathways through interactions with Eph receptors [6], TRAF6-dependent TLR4 signaling complexes [2], Gαs-dependent signaling [23], and β-arrestin-mediated receptor networks [22]. Downstream, it associates with proteins that regulate actin dynamics, cell adhesion, and migration, including IQGAP1, cortactin, CRKL, and β-arrestin 1. Particularly notable is IQGAP1, a central organizer of receptor signaling, small GTPases, adhesion complexes, and the actin cytoskeleton. Together, these interactions position SASH1 to translate extracellular cues into coordinated changes in signaling, cytoskeletal organization, and cellular behavior.

This organizational logic resembles that of multidomain scaffold proteins such as the SHANK family, which couples receptor-associated signaling complexes to cytoskeletal regulatory machinery [80,81,82,83]. Notably, SHANK-containing postsynaptic scaffolds assemble signaling condensates through phase separation that cluster receptor components and promote actin assembly [84], providing a mechanistic precedent for the phase-separation hypothesis raised above. Although SASH1 differs substantially in biological context and molecular composition, both illustrate how non-catalytic scaffold proteins can generate diverse cellular outputs through network organization rather than enzymatic activity. Importantly, SASH1 extends this principle through interactions with multiple scaffold and adaptor proteins—including IQGAP1, tankyrase-2, β-arrestin 1, cortactin, and CRKL—creating a hierarchical ‘scaffold-of-scaffolds’ architecture capable of integrating multiple signaling systems.

We therefore propose that the principal function of SASH1 is not enzymatic catalysis but the organization of signaling architecture. As a context-dependent scaffold, SASH1 integrates receptor-proximal signals with downstream signaling and cytoskeletal networks, providing a unifying explanation for its pleiotropic biological functions. This model further suggests that disease-associated SASH1 variants may act by disrupting the assembly, regulation, or specificity of these signaling networks rather than by impairing a single downstream pathway.

## 5. SASH1-Associated Pigmentary Diseases (SAPD) Reveal Structure–Function Relationships in SASH1

### 5.1. Human SAPD Provides Genetic Evidence for SASH1 Function

Pigmentation disorders provide the strongest evidence linking SASH1 molecular architecture to physiological function. Unlike most pigmentation genes, which encode enzymes, transporters, or transcription factors [85], SASH1 encodes a multidomain scaffold protein. Germline SASH1 variants cause several Mendelian pigmentary disorders, providing a unique opportunity to connect specific protein-interaction surfaces with human phenotype [5,28,45]. Pathogenic SASH1 variants have been identified in multiple families with lentiginous phenotypes, dyschromatosis universalis hereditaria, reticulate pigmentation disorders, and related syndromes characterized by mixed hyperpigmented and hypopigmented lesions [8,23,28,29,30,31,32,33,34,36,37]. More recently, SASH1 variants have also been linked to premature hair graying by disrupting melanocyte stem-cell maintenance [5]. These are rare Mendelian conditions, reported to date in individual families and small pedigrees rather than at appreciable population frequency; inheritance is typically autosomal dominant with high penetrance. The recurrence of variants clustering at the conserved SPIDER-region hotspot across unrelated families reinforces a direct genotype–phenotype relationship [28].

These disorders are particularly informative because they arise from inherited alterations of a signaling organizer rather than a classical pigment-producing enzyme. Consequently, SAPD provides a natural human genetic system for defining how specific structural elements of a scaffold protein control cellular behavior and tissue homeostasis.

### 5.2. Pigmentation Variants Identify a Functional Interaction Hub

A striking feature of SAPD is that pathogenic variants are not randomly distributed across SASH1. Instead, they cluster within the highly conserved SPIDER–SH3–SAM1 region, which contains the majority of currently characterized protein-interaction surfaces [5,28,45]. More than 70% of reported pigmentation-associated variants localize to the SPIDER, particularly around the tandem tankyrase-binding motifs (TBM) TBM6 and TBM7 [5,28], whereas approximately 20% occur within the SH3 domain (Figure 4). Only a small number have been identified within SAM1, and none have been reported in SAM2 or the long C-terminal linker (Figure 4).

This non-random distribution strongly argues that pigmentation phenotypes arise from perturbation of specific molecular interactions rather than from complete loss of SASH1 expression or global protein destabilization. Consistent with this idea, most SAPD variants are missense substitutions predicted to alter interaction surfaces while preserving overall protein structure.

Among currently known SASH1 interactions, the strongest structure–function relationship links pigmentation phenotypes to tankyrase 2 (TNKS2). The SPIDER contains four functional tankyrase-binding motifs that engage TNKS2 through multivalent interactions [5]. The best-characterized example is S519N, which occurs within TBM6 and alters TNKS2 binding affinity without abolishing the interaction [5]. Despite this modest biochemical effect, S519N disrupts melanocyte stem-cell maintenance and causes premature hair graying, demonstrating how subtle changes in scaffold engagement can produce clinically significant phenotypes.

The second example is R644W within the SAM1 region [45]. This arginine is conserved at a position required for self-association of the homologous SASH3 SAM domain; however, the R644W substitution did not measurably alter the monomer–oligomer equilibrium of SASH1 SAM1 in vitro, and its pathogenic mechanism therefore remains unresolved.

Additional variants further support the importance of the central SPIDER–SH3–SAM1 region. Y551D, located adjacent to TBM7, identifies the TBM6–TBM7 segment as a recurrent mutational hotspot [5,28,86]. The recessive E617K variant, located immediately C-terminal to the SH3 domain, causes a syndromic phenotype that includes palmoplantar keratoderma and increased susceptibility to cutaneous carcinoma [29]. Although its pathogenic mechanism remains unclear, its localization further supports the SPIDER–SH3–SAM1 region as a major functional hotspot within SASH1.

Collectively, the genetic architecture of SAPD identifies the SPIDER–SH3–SAM1 region as a critical signaling hub and implicates altered SASH1–TNKS2 interactions as a major determinant of pigmentation pathology.

### 5.3. Mechanistic Insights into Pigmentation Regulation

Current evidence supports a model in which SASH1 regulates pigmentation by organizing signaling complexes that control melanocyte homeostasis. The strongest experimental support exists for TNKS2-dependent regulation of melanocyte stem-cell maintenance. The clearest mechanistic link comes from the S519N variant, which alters TNKS2 binding and impairs melanocyte stem-cell maintenance without eliminating the interaction [5]. Together with the clustering of disease-associated variants around tankyrase-binding motifs, these findings establish a direct connection between SASH1 molecular architecture, TNKS2 complex assembly, and pigmentation phenotypes. Importantly, disease appears to result from quantitative disruption of a specific scaffold interaction rather than complete loss of protein activity.

SASH1 has also been implicated in additional pathways relevant to pigmentation. A Gαs–SASH1–IQGAP1–E-cadherin signaling axis has been proposed to regulate melanocyte migration [23], while SASH1 promotes melanogenesis and melanocyte migration through modulation of TGF-β1 signaling [37]. SASH1 has also been suggested to participate in a p53–POMC–Gαs–SASH1 positive-feedback loop that amplifies ultraviolet (UV)-induced melanogenic responses [36]. Together, these studies indicate that SASH1 influences multiple aspects of melanocyte biology, including stem-cell maintenance, migration, adhesion, and pigment production.

Whether these additional pathways are coordinated through TNKS2-containing complexes or represent independent SASH1 signaling networks remains unknown. Thus, although TNKS2-dependent regulation of melanocyte stem cells [5,87] currently provides the strongest mechanistic framework, SASH1 likely functions more broadly as a signaling organizer that integrates multiple pathways controlling melanocyte behavior.

In summary, pigmentation disorders provide the strongest genotype-to-phenotype evidence for SASH1 function. The clustering of pathogenic variants within the interaction-rich SPIDER–SH3–SAM1 region, together with mechanistic studies of TNKS2-dependent regulation of melanocyte stem cells, establishes a direct link between SASH1 molecular architecture and human disease. These findings provide a framework for understanding how disruption of specific SASH1 interaction networks produces distinct biological outcomes.

## 6. SASH1 in Cytoskeletal Dynamics, Cell Adhesion, and Migration

Among the diverse biological processes associated with SASH1, regulation of cell adhesion and migration is one of the most consistently observed. Across diverse cell types and disease contexts, SASH1 has been implicated in processes requiring dynamic remodeling of the actin cytoskeleton, including cell adhesion, lamellipodia formation, migration, and epithelial–mesenchymal transition (EMT) [1,3]. Although the underlying mechanisms remain incompletely understood and these activities have often been studied independently, the available data support a model in which SASH1 acts as a signaling organizer that links extracellular cues to cytoskeletal remodeling machinery.

### 6.1. Actin Remodeling Through Cortactin

SASH1 was first linked to the actin cytoskeleton through its interaction with cortactin [1]. Cortactin is a key regulator of actin polymerization and is enriched in lamellipodia and other membrane protrusions involved in cell migration [76,77]. SASH1 co-localizes with cortactin in F-actin-rich lamellipodia, and its conserved SH3–SAM1 region contributes to targeting SASH1 to these structures [1]. The binding interface itself was not mapped in that study, however: only full-length SASH1 co-precipitated cortactin, whereas the isolated SH3–SAM1 region and the two other deletion constructs tested did not, indicating that the structural basis of the interaction is more complex than a single contiguous region. These findings also leave open the possibility that the observed association is not mediated solely by a direct SH3-SAM1–cortactin interaction but may require additional SASH1 domains or other components of a larger protein complex. Functional studies demonstrated that SASH1 expression influences cell migration and cell–matrix adhesion, supporting a role for SASH1 in processes that require dynamic cytoskeletal remodeling [1]. These findings suggest that SASH1 is positioned at sites of active actin reorganization, where it may coordinate signaling events that regulate cell movement.

### 6.2. Integration of Signaling with Cytoskeletal Scaffold Networks Through IQGAP1

SASH1 also interacts with IQGAP1 [23]. IQGAP1 is a multifunctional scaffold that integrates signals from numerous pathways controlling actin dynamics, cell adhesion, and migration [66]. Unlike cortactin, which functions directly within actin-rich protrusions, IQGAP1 serves as a signaling hub that organizes protein complexes at the interface between extracellular signals and cytoskeletal responses. Silencing IQGAP1 reverses the loss of E-cadherin caused by disease-associated SASH1 variants [23], and this association places SASH1 within a broader network of scaffold proteins that regulate cellular architecture and motility.

Together, the interactions of SASH1 with cortactin and IQGAP1 support a model in which SASH1 contributes to the organization of signaling complexes associated with the actin cytoskeleton. Importantly, both binding partners are linked to pathways that regulate cell adhesion, migration, and cytoskeletal remodeling, cellular processes that are repeatedly affected by altered SASH1 expression in cancer and other biological contexts. While direct mechanistic links remain to be established, these observations support that modulation of cytoskeletal organization and cell motility represents a core function of SASH1.

## 7. SASH1 in Receptor-Mediated Signaling

Another recurring theme across diverse SASH1-associated pathways is its role in downstream of cell-surface receptors, where it serves as a scaffold linking receptor activation to intracellular signaling networks. Through its multidomain architecture, SASH1 engages signaling adaptors, kinases, and cytoskeletal regulators, enabling the assembly of pathway-specific complexes. These studies provide strong support for the unified model of SASH1 as a signaling organizer that translates extracellular cues into coordinated cellular responses.

### 7.1. SASH1 in TLR4 Signaling

The best-characterized example of SASH1-mediated signal transduction is the Toll-like receptor 4 (TLR4) pathway in endothelial cells. Following lipopolysaccharide (LPS) stimulation, SASH1 binds TRAF6 through a conserved TRAF6-binding motif and independently associates with TAK1 and IKKβ, promoting assembly of a TRAF6–TAK1–IKKβ signaling complex [2]. Loss of SASH1 impairs NF-κB activation and inflammatory cytokine production, whereas increased SASH1 expression enhances both responses. These findings establish SASH1 as a bona fide signaling scaffold that couples receptor activation to downstream transcriptional programs.

### 7.2. SASH1 in EphA8 Signaling

SASH1 also participates in receptor tyrosine kinase signaling through direct interaction with Eph receptors. Structural and biochemical studies identified SASH1 as a novel Eph receptor-binding partner that engages the Eph receptor SAM domain through its SAM1 domain, with the highest affinity observed for EphA8. The crystal structure of the EphA8–SASH1 complex defined the molecular basis of this interaction, and cellular studies demonstrated that SASH1 promotes EphA8 kinase activation in a SAM-dependent manner [6]. These findings establish SASH1 as a direct regulator of Eph receptor signaling and provide a mechanistic link between SASH1 and receptor systems involved in cell adhesion, migration, and tissue organization.

### 7.3. SASH1 in β-Arrestin-Mediated Signaling

Additional evidence linking SASH1 to receptor-associated signaling comes from its interaction with β-arrestin 1, which is best known as a multifunctional adaptor that coordinates signaling downstream of many G protein-coupled receptors (GPCRs) [71,72,73,74,75]. This pathway highlights SASH1 as a component of a β-arrestin-dependent signaling complex that regulates endothelial function and lung development [22].

Coulombe and colleagues identified SASH1 as a β-arrestin 1 binding partner and showed that the interaction is mediated by the SASH1 SAM1 domain [22]. In developing lung endothelial cells, SASH1 is required for β-arrestin 1-dependent activation of the Akt–eNOS pathway. Loss of either β-arrestin 1 or SASH1 reduces Akt activation and eNOS phosphorylation; loss of SASH1 additionally reduces nitric oxide production and disrupts normal alveolar maturation. These findings establish SASH1 as an essential component of a receptor-associated signaling pathway controlling endothelial development.

Although this β-arrestin 1-dependent developmental function was mapped downstream of TLR4 using LPS stimulation in vitro, the endogenous ligand or developmental signal responsible for activating this pathway remains undefined. Nevertheless, the study provides strong evidence that SASH1 functions within β-arrestin-mediated signaling networks. Importantly, it also represents one of the clear domain-specific functions assigned to SASH1, linking the SAM1 domain to assembly of a physiologically relevant signaling complex.

Collectively, the TLR4, EphA8, and β-arrestin studies indicate that SASH1 participates in signaling downstream of multiple receptor classes, including innate immune receptors, receptor tyrosine kinases, and potentially GPCR-associated pathways. Despite the diversity of these systems, a common mechanistic theme emerges: SASH1 functions as a modular scaffold that organizes signaling complexes and couples extracellular stimuli to downstream cellular and transcriptional responses.

## 8. SASH1 in Cancer: Context-Dependent Functions

### 8.1. Evidence for a Tumor-Suppressive Role

SASH1 was originally identified as a candidate tumor suppressor through loss-of-heterozygosity analysis of chromosome 6q24.3 in breast cancer [7]. Subsequent studies reported reduced SASH1 expression or tumor-suppressor activity in multiple malignancies, including breast [13], lung [14], colorectal [3], melanoma [18], glioma [17], osteosarcoma [19], gastric [16], and head and neck squamous cell carcinoma [88], with lower expression frequently correlating with advanced disease stage, lymph-node involvement, metastasis, and poorer survival. These observations established SASH1 as a potential prognostic biomarker and suggested that SASH1 loss contributes to tumor progression.

Unlike the inherited pigmentary disorders discussed above, where germline variants directly link SASH1 structure to disease, evidence in cancer primarily comes from altered expression and functional restoration studies. Collectively, these studies support a predominantly tumor-suppressive role for SASH1 while also revealing substantial context dependence. It should be noted, however, that most of these findings are correlative. Although reduced SASH1 expression is frequently associated with poor clinical outcome, a driver role for SASH1 loss has been demonstrated in only a subset of experimental models and should not be assumed across all cancer types.

### 8.2. SASH1 Suppresses EMT and Metastasis Through Cytoskeletal and Adhesion Signaling

The cytoskeletal and adhesion functions discussed above have direct implications for cancer progression. Because EMT, invasion, and metastasis require extensive remodeling of adhesion and actin networks, SASH1-dependent control of these processes is likely a major mechanism underlying its tumor-suppressive activity on tumor invasion and metastasis. In colorectal cancer, SASH1 suppresses EMT through interaction with the adaptor protein CRKL. Binding of the SASH1 PxxP motif to the CRKL SH3 domain competitively disrupts pro-migratory CRKL signaling and reduces EMT-associated phenotypes [3]. This interaction provides one of the clearest examples of a defined molecular mechanism linking SASH1 structure to tumor suppression.

Additional studies support anti-invasive functions in other tumor types. SASH1 inhibits proliferation of melanoma cells via G2/M cell-cycle arrest [18], reduces invasiveness in glioma [17], and suppresses proliferation and metastatic behavior in osteosarcoma [19]. These findings are consistent with evidence that SASH1 localizes to F-actin-rich lamellipodia through interactions involving cortactin and other cytoskeletal regulators [1], positioning SASH1 to influence cell adhesion, migration, and cytoskeletal remodeling.

Viewed through the structure–function framework developed in this review, SASH1 suppresses metastasis by coordinating both cytoskeletal and signaling networks. Loss of SASH1 simultaneously weakens adhesion-associated complexes, enhances EMT-promoting pathways, and reduces apoptotic sensitivity, creating a cellular state favorable for invasion and dissemination.

### 8.3. SASH1 Regulates Proliferation and Apoptosis

Evidence also suggests the ability of SASH1 to restrain cell growth and promote apoptosis. Restoration of SASH1 expression suppresses PI3K/Akt signaling, reducing Akt phosphorylation and slowing proliferation in cutaneous squamous cell carcinoma cells [20]. SASH1 also enhances apoptotic responses. In breast cancer cells, increased SASH1 expression promotes caspase-3-dependent cell death [4,40].

Mechanistically, apoptosis is linked to one of the best-characterized post-translational switches in SASH1 biology. Caspase-3 cleaves SASH1 at Asp230, generating a large C-terminal fragment encompassing residues 231–1247. This fragment translocates to the nucleus and promotes NF-κB activation, establishing a positive-feedback loop that amplifies apoptotic signaling [4]. Thus, proteolytic processing converts SASH1 from a predominantly cytoplasmic scaffold into a nuclear signaling factor, illustrating how structural remodeling can fundamentally alter SASH1 function.

### 8.4. Context-Dependent Functions and the Limits of the Tumor-Suppressor Model

Despite substantial evidence supporting tumor-suppressive activity, SASH1 does not behave uniformly across all cancer types. In breast cancer, prognostic associations vary markedly by molecular subtype. High SASH1 expression predicts favorable outcomes in low-grade estrogen receptor-positive tumors but correlates with significantly poorer relapse-free survival in HER2-positive breast cancer, with a similar but non-significant trend in triple-negative disease [40]. In HER2-positive disease, elevated SASH1 expression was associated with a hazard ratio of 3.07 for relapse, challenging the notion that SASH1 functions exclusively as a tumor suppressor.

The scaffold model readily explains these observations. Unlike enzymes, scaffold proteins do not possess a single intrinsic biochemical activity. Instead, their functional output depends on the signaling complexes they assemble. In some settings, SASH1 primarily recruits anti-tumor effectors, including CRKL-regulatory and pro-apoptotic complexes. In others, SASH1 may facilitate signaling pathways that support tumor survival, particularly those involving NF-κB-dependent inflammatory programs. The balance among these outputs is likely determined by cell type, signaling environment, post-translational modifications, and the relative abundance of interacting partners.

Rather than functioning as a uniformly tumor-suppressive or oncogenic factor, SASH1 appears to act as a context-dependent signaling scaffold whose biological effects depend on the signaling complexes assembled in a particular tumor type and microenvironment.

## 9. SASH1 in Vascular Biology

### 9.1. Endothelial Function

The receptor-mediated signaling activities of SASH1 have important consequences for vascular function and endothelial homeostasis. Endothelial SASH1 is required for normal lung alveolar maturation, as genetic disruption of *Sash1* impairs this process in mice by disrupting β-arrestin 1-dependent Akt–eNOS signaling in endothelial cells [22]. The resulting defect is perinatally lethal: *Sash1*-null pups are born cyanotic and die of respiratory failure immediately after birth, their lungs failing to complete the alveolar maturation required for postnatal gas exchange. These findings establish a functional requirement for SASH1 in endothelial-dependent lung maturation and demonstrate that SASH1 contributes to endothelial nitric oxide signaling in vivo. This developmental role has so far been demonstrated only in lung maturation; given the close physiological coupling of the pulmonary and cardiovascular systems, endothelial SASH1–eNOS signaling may have broader vascular relevance.

In addition to its developmental role, SASH1 has emerged as a negative regulator of angiogenesis. Yan and colleagues identified SASH1 as a downstream target of miR-128-3p and showed that its exosomal delivery promotes endothelial migration, proliferation, and tube formation [35]. Independently, SASH1 silencing in human aortic endothelial cells increases migration, proliferation, and Matrigel tube formation [9], implicating SASH1 as a negative regulator of angiogenesis. These findings suggest that post-transcriptional regulation of SASH1 is an important mechanism controlling endothelial activation and angiogenic capacity.

### 9.2. Vascular Disease

Several studies have implicated SASH1 in vascular pathology. SASH1 expression is markedly elevated in the atherosclerotic carotid arteries of smokers compared with non-smokers, suggesting a role in smoking-associated vascular remodeling [9]. Whether this increase reflects a protective response to vascular injury or contributes directly to disease progression remains unknown. Given SASH1’s established roles in endothelial signaling and inflammatory pathways, both possibilities remain plausible.

SASH1 has also been implicated in pre-eclampsia, a pregnancy-associated vascular disorder characterized by endothelial dysfunction and impaired placental vascularization. SASH1 expression is increased in pre-eclamptic placentas, and experimental overexpression suppresses trophoblast proliferation and invasion while promoting apoptosis [12]. These effects are consistent with the anti-migratory and anti-angiogenic activities attributed to SASH1 in other cellular systems and suggest that dysregulated SASH1 expression may contribute to abnormal placental vascular remodeling.

Although the underlying mechanisms remain incompletely defined, current evidence indicates that SASH1 lies at the intersection of endothelial function, angiogenesis, and vascular disease. Future studies will be needed to determine whether SASH1 primarily serves a protective homeostatic role or actively contributes to vascular pathology and whether modulation of SASH1 expression represents a potential therapeutic strategy in vascular disorders.

## 10. Regulation of SASH1 Expression and Activity

Because SASH1 lacks intrinsic catalytic activity, regulation of its abundance, localization, and interaction state is likely a major determinant of function. However, compared with the expanding literature on SASH1 biology, surprisingly little is known about the mechanisms that control SASH1 expression and activity.

SASH1 is broadly expressed across human tissues while being largely absent from lymphocytes, where the related SLy-family protein SLY1/SASH3 predominates [27]. Despite its diverse biological functions, relatively little is known about how SASH1 expression is regulated. SASH1 expression has been shown to be induced during the UV-responsive pigmentation pathway downstream of p53–POMC–α-MSH signaling [36] and is elevated in the atherosclerotic vasculature of smokers [9]. In addition, the antihistamine chloropyramine increases SASH1 expression in breast cancer cells [40]. Post-transcriptional regulation also contributes to SASH1 abundance, as both miR-21 [89] and miR-128-3p [35] directly target and suppress SASH1, the latter promoting endothelial migration and angiogenesis. Beyond these examples, the transcriptional and epigenetic mechanisms controlling SASH1 expression remain largely unknown. Post-translational modification of SASH1 is also important for its activity, as discussed in Section 4.4.

## 11. Knowledge Gaps in Structure–Function Relationships and Future Directions

Despite growing evidence that SASH1 functions as a multi-docking signaling hub, major gaps remain in understanding how its molecular architecture and regulation give rise to its diverse biological functions. The most significant limitation is the lack of experimental structures encompassing multiple SASH1 domains. Although AlphaFold2 predictions provide valuable hypotheses [44], no structures are currently available for the SPIDER–SH3–SAM1 tri-domain region or for full-length SASH1. Consequently, many aspects of SASH1 regulation and structure–function relationships remain unresolved. In addition, despite evidence that phosphorylation [61] and tankyrase-2 binding [5] may regulate SASH1 activity, localization, and protein interactions, systematic studies of SASH1 post-translational modifications (PTMs) are largely lacking.

In addition, several key mechanistic questions remain unresolved. These include whether SAM1-binding partners engage overlapping or distinct interaction surfaces; whether ligand-induced SAM1 folding occurs through conformational selection or induced fit; whether SAM2 has a physiological binding partner; whether the AlphaFold2-predicted SAM-like helical bundle adjacent to SAM2 represents a folded structure in cells; and how full-length SASH1 is organized under different signaling conditions, including whether multiple interaction surfaces can be engaged simultaneously.

An important priority for future studies is to move beyond isolated domains toward an integrated understanding of SASH1 architecture and regulation. In particular, it will be important to determine whether the conserved SPIDER–SH3–SAM1 region functions as an interconnected supra-domain that coordinates receptor signaling, adaptor recruitment, and cytoskeletal regulation. Equally important is defining how PTMs control SASH1 conformational states, interaction networks, and signaling outputs. Addressing these questions will require a combination of structural, proteomic, and cell biological approaches, including NMR, cryo-EM, cross-linking mass spectrometry, quantitative PTM mapping, proximity-labeling interactomics, and systematic analysis of disease-associated variants. Such studies should establish how SASH1 assembles context-dependent signaling complexes and how disruption of these assemblies contributes to human disease.

More broadly, SASH1 offers an opportunity to investigate fundamental principles governing multidomain scaffold proteins, including how intrinsically disordered regions cooperate with structured domains, how post-translational modifications remodel interaction networks, and how disease-associated variants perturb signaling architecture. Insights gained from SASH1 may therefore inform the study of many other scaffold proteins whose functions are similarly encoded through dynamic protein–protein interaction networks.

## 12. Conclusions

Accumulated structural, biochemical, and functional evidence supports a unifying model in which SASH1 operates as a context-dependent scaffold that integrates receptor-proximal signals with intracellular signaling pathways and cytoskeletal dynamics. Its multidomain architecture—combining intrinsically disordered regions, modular interaction motifs, and dual SAM domains—enables the assembly of distinct, context-dependent signaling complexes across diverse cell types, thereby explaining its pleiotropic roles in pigmentation, vascular biology, immunity, and cancer. This framework not only reconciles previously disparate observations but also highlights how disease-associated variants likely perturb the strength, composition, or regulation of specific SASH1 interactions within multi-partner complexes. Moving forward, resolving full-length structures, defining post-translational regulation, and mapping dynamic interactomes will be critical for understanding how SASH1 coordinates signaling architecture in health and disease and for evaluating its potential as a therapeutic target.

Beyond the specific roles highlighted in this review, SASH1 serves as a compelling model for understanding how non-catalytic proteins organize cellular signaling networks. The integration of structural biology, human genetics, and interactome analyses in SASH1 research provides a unique opportunity to directly connect molecular architecture with physiological and pathological outcomes. As interest in scaffold proteins increases, both as determinants of signaling specificity and as potential therapeutic targets, SASH1 represents a valuable system for defining general principles by which multidomain interaction hubs regulate cellular behavior.

## Figures and Tables

**Table 2:** SASH1 disease associations.

Disease Category	Specific Disease	SASH1 Status	Key Finding
Hereditary pigmentation disorders	Lentiginous phenotypes; premature hair graying, dyschromatosis universalis hereditaria; recessive keratoderma–skin carcinoma syndrome	Germline variants (mostly autosomal-dominant missense; rare recessive)	Variants alter melanocyte stem-cell maintenance and possibly melanogenesis, producing mixed hyper- and hypopigmentation and premature hair graying [5,8,23,28,29,30,31,32,33,34]
Vascular/cardiovascular	Atherosclerosis (smoking-associated); pre-eclampsia	Upregulated	Proposed link between smoking and atherosclerosis [9]; intersects eNOS dysregulation [22]; suppresses trophoblast proliferation/invasion [12]
Cancer	Breast, colorectal, lung (NSCLC), gastric, glioma, melanoma, osteosarcoma	Most downregulated (tumor suppressor)	Reduced SASH1 correlates with invasion, metastasis, and poorer survival; restoring SASH1 (e.g., chloropyramine) suppresses malignant phenotypes [3,13,14,15,40]
Neurological/injury	Spinal-cord injury; glioma	Induced in reactive astrocytes (SCI); reduced in glioma	SASH1 depletion reduces reactive astrocytosis and improves functional recovery after spinal-cord injury [38]; SASH1 overexpression reduces glioma-cell invasion [17]
Other genetic associations	Alzheimer’s disease [10]; diabetic nephropathy [11]	Genetic/expression associations	Epidemiological and expression links reported; mechanism not yet characterized

NSCLC, non-small-cell lung cancer; SCI, spinal-cord injury

**Table 3:** Location, structure, and function of regions and domains in SASH1.

Region/Domain	Location (Residues)	Molecular Structure	Molecular Function	Physiological Function
NLS1	259–275	Predicted disordered	Nuclear import [3]	Unknown
NLS2	452–468 (within SPIDER)	Predicted disordered	Nuclear import [3]	Unknown
SPIDER	~400–554	Intrinsically disordered [41]	Scaffold for tankyrase-2 (TNKS2) via tankyrase-binding motifs [5,43]	Pigmentation (multiple variants)
SH3	555–615	Folded SH3 β-barrel; PDB 2EBP	Binds proline-rich ligands (partners not yet defined)	Targets SASH1 to F-actin-rich lamellipodia with cortactin [1]; pigmentation
SAM1	633–697	Conformationally heterogeneous: disordered monomer ⇌ folded helical bundle; folds on binding [6,45]	Binds EphA8 SAM domain [6]; Caskin1 tandem SAM [21]; β-arrestin 1 [22]	Pigmentation; eNOS/NO signaling
TRAF6-binding motif	852–860 (DVPTEVTEP)	Predicted disordered	Bound by TRAF6 [2]	NF-κB activation in endothelial cells downstream of TLR4
PxxP	985–990 (PPVPAK in UniProt)	Predicted disordered	Binds SH3-N domain of CRKL [3]	Inhibits CRKL signaling and EMT downstream of RTK
SAM2	1177–1241	Well-folded, monomeric five-helix SAM fold; PDB 2DL0 [42]	No binding partners identified	Unknown

**Table 4:** Structural and molecular features underlying SASH1 multifunctionality.

Structural Feature	Key Characteristics
Modular, separable interaction surfaces	Composed of multiple discrete domains and motifs, each capable of engaging distinct binding partners (e.g., tankyrase-2, EphA8, β-arrestin 1, TRAF6, CRKL)
Intrinsic disorder and conformational plasticity	The SPIDER is intrinsically disordered (NMR-validated), and the SAM1 domain exhibits structural flexibility, enabling adaptable interactions
Dual SAM domains	Contains two SAM domains (SAM1 and SAM2); SAM2 is unique to SASH1 and absent in other SLy family members
Post-translational regulation	Function is modulated by caspase cleavage and phosphorylation
Scaffold-of-scaffolds	Functions as a higher-order hub by binding other scaffold proteins, including IQGAP1, β-arrestin 1, cortactin, and CRKL

## Data Availability

No new data were created or analyzed in this study. Data sharing is not applicable to this article.

## Abbreviations

The following abbreviations are used in this manuscript:

α-MSH	α-melanocyte-stimulating hormone
AP-MS	affinity purification–mass spectrometry
ARC4	ankyrin-repeat cluster 4
co-IP	co-immunoprecipitation
CRKL	CRK-like proto-oncogene (adaptor protein)
EMT	epithelial–mesenchymal transition
eNOS	endothelial nitric oxide synthase
EphA8	ephrin type-A receptor 8
GPCR	G-protein-coupled receptor
IGF-1	insulin-like growth factor 1
IKK	IκB kinase
IQGAP1	IQ-motif-containing GTPase-activating protein 1
LLPS	liquid–liquid phase separation
LPS	lipopolysaccharide
ML/EH	mid-loop/end-helix (SAM-domain interaction surfaces)
MSCP	multi-SAM-containing protein
NF-κB	nuclear factor-κB
NLS	nuclear localization signal
NMR	nuclear magnetic resonance
NSCLC	non-small-cell lung cancer
PARP	poly(ADP-ribose) polymerase
PDB	Protein Data Bank
PI3K	phosphoinositide 3-kinase
PKM2	pyruvate kinase M2
PMA	phorbol 12-myristate 13-acetate
POMC	pro-opiomelanocortin
PTM	post-translational modification
PxxP	proline-rich motif
RTK	receptor tyrosine kinase
SAM	sterile alpha motif
SAM1	first SAM domain of SASH1
SAM2	second SAM domain of SASH1
SAPD	SASH1-associated pigmentary diseases
SCI	spinal-cord injury
SH2	SRC-homology-2 domain
SH3	SRC-homology-3 domain
SLy	SH3-domain protein expressed in lymphocytes
SPIDER	SLy Proteins Associated Disordered Region
TAK1	TGF-β-activated kinase 1
TBI	traumatic brain injury
TBM	tankyrase-binding motif
TGF-β	transforming growth factor-β
TLR4	Toll-like receptor 4
TNKS2	tankyrase-2
TRAF6	TNF receptor-associated factor 6
UV	ultraviolet
Y2H	yeast two-hybrid
